# Silica Waveguide Thermo-Optic Mode Switch with Bimodal S-Bend

**DOI:** 10.3390/nano14241991

**Published:** 2024-12-12

**Authors:** Zhentao Yao, Manzhuo Wang, Yue Zhang, Zhaoyang Sun, Xiaoqiang Sun, Yuanda Wu, Daming Zhang

**Affiliations:** 1 State Key Laboratory of Integrated Optoelectronics, College of Electronic Science & Engineering, Jilin University, No. 2699 Qianjin Street, Changchun 130012, China; yaozt22@mails.jlu.edu.cn (Z.Y.); mzwang24@mails.jlu.edu.cn (M.W.); zyue22@mails.jlu.edu.cn (Y.Z.); szy22@mails.jlu.edu.cn (Z.S.); zhangdm@jlu.edu.cn (D.Z.); 2 College of Materials Science and Opto-Electronic Technology, University of Chinese Academy of Sciences, Beijing 100049, China; wuyuanda@semi.ac.cn; 3 Key Laboratory of Optoelectronic Materials and Devices, Institute of Semiconductors, Chinese Academy of Sciences, Beijing 100083, China

**Keywords:** thermo-optic, silica waveguide, mode switch, integrated optics

## Abstract

A silica waveguide thermo-optic mode switch with small radius bimodal S-bends is demonstrated in this study. The cascaded multimode interference coupler is adopted to implement the E_11_ and E_21_ mode selective output. The beam propagation method is used in design optimization. Standard CMOS processing of ultraviolet photolithography, chemical vapor deposition, and plasma etching are adopted in fabrication. Detailed characterizations on the prepared switch are performed to confirm the precise fabrication. The measurement results show that within the wavelength range from 1530 to 1575 nm, for the E_11_ mode input, the switch exhibits an extinction ratio of ≥13.1 dB and a crosstalk ≤−22.8 dB at an electrical driving power of 284.8 mW, while for the E_21_ mode input, the extinction ratio is ≥15.5 dB and the crosstalk is ≤−18.1 dB at an electrical driving power of 282.4 mW. These results prove the feasibility of multimode S-bends in mode switching. The favorable performance of the demonstrated switch promises good potential for on-chip mode routing.

## 1. Introduction

To meet the demand on the wide bandwidth (BW) and large capacity transmission in optical networks, various multiplexing technologies have been explored in recent years. Among them, the mode division multiplexing (MDM) technology that utilizes orthogonal optical mode channels offers an additional freedom of data transmission except for wavelength division multiplexing (WDM) and polarization division multiplexing (PDM) [1,2,3]. Different functional modules, including a mode multiplexer/demultiplexer [4,5,6], bending waveguide [4,7,8], 3 dB coupler [9,10], and optical switches have been adopted in the network construction [11,12,13,14]. For reconfigurable mode routing, the mode switch plays an important role. In Ref. [11], the asymmetric Y-junction with a multi-mode interference (MMI) coupler is used to conduct the selective mode switching. A cascaded Mach–Zehnder interferometer (MZI) has also been proposed to allow the pass or cutoff of four modes [12]. The micro-ring resonator-based switch has been proven to be useful in MDM-WDM complex multiplexing [13]. However, most of these demonstrated switches are only effective for the fundamental mode [14]. The direct mode routing for high-order modes is yet to be investigated. Due to the merits of broadband transparency, long-term stability, low-loss coupling to glass fibers, and compatibility with complementary metal oxide semiconductor (CMOS) fabrication, silica waveguide have been adopted in the construction of mode switching [15].

In this work, a silica waveguide thermo-optic mode switch is theoretical designed and experimentally demonstrated. Cascaded 1 × 2 and 2 × 2 MMI couplers are adopted to implement the E_11_ and E_21_ mode selective output. Small-radius bimodal S-bends based on gap waveguides are utilized for compact design. The beam propagation method (BPM) is used in design optimization. The measurement results show that within the wavelength range from 1530 to 1575 nm, for the E_11_ mode input, the extinction ratio (ER) is ≥13.1 dB and the crosstalk (CT) is ≤−22.8 dB at the electrical driving power of 284.8 mW. Meanwhile, for the E_21_ mode input, ER is ≥ 15.5 dB and CT is ≤−18.1 dB at the electrical driving power of 282.4 mW. The measured rise time and fall time are 1.04 ms and 1.32 ms, respectively. The demonstrated switch offers a solution for selective mode routing, which holds favorable potential in MDM applications.

## 2. Design and Optimization

**Figure 1:**
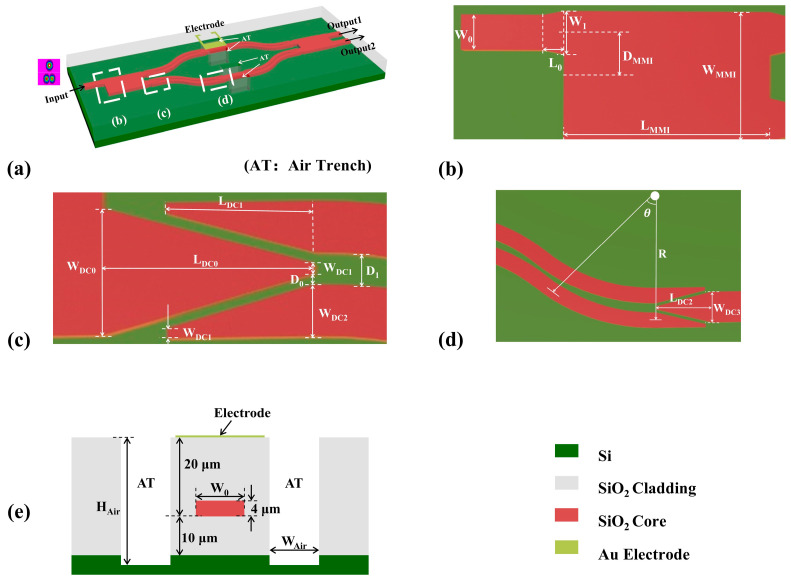
Schematic diagram of (**a**) three-dimensional view of proposed thermo-optic mode switch, and the geometric size of (**b**) E_11_-E_21_ dual-mode MMI coupler, (**c**) input end, (**d**) output end of E_11_-E_21_ dual-mode S-bend, and (**e**) cross-sectional view of the dual-mode modulation arm with metal phase shifter.

**Figure 2:**
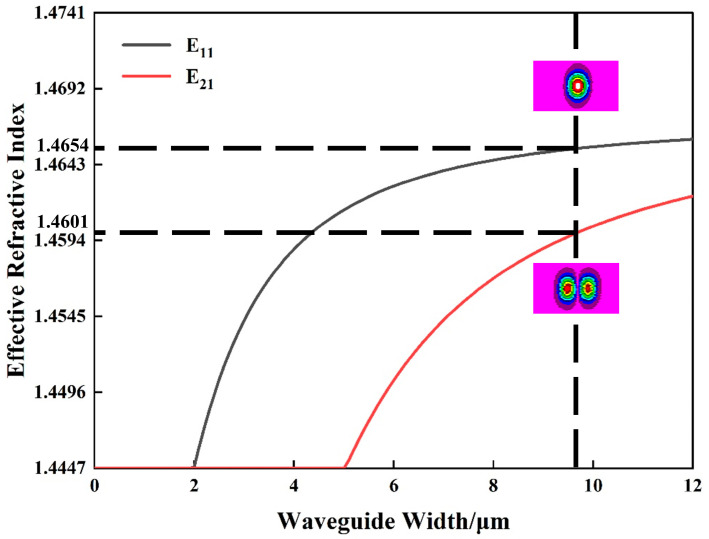
Effective refractive index as a function of waveguide width at *λ* = 1550 nm.

The proposed silica waveguide dual-mode thermo-optic switch is shown in Figure 1a. In this 1 × 2 switch configuration, the dual-mode MMI could implement the function of 3 dB beam splitting and a combination of E_11_ and E_21_ modes, as shown in Figure 1b. Two straight multimode waveguides that support the propagation and modulation of E_11_ and E_21_ modes connect two input/output ports of the MMI coupler. The dual-mode gap shape S-bends that connect the MMI coupler and the straight waveguide are optimized by BPM to allow the low-loss propagation of E_11_ and E_21_ modes. The geometric size and shape of tapered waveguides, horn waveguides, and the gap are finely customized, as shown in Figure 1c,d. The cross-sectional view of the dual-mode modulation arm with a metal phase shifter is demonstrated in Figure 1e. The refractive index of both the upper and lower claddings is 1.4447. Meanwhile, the refractive index of the germanium-doped silica core layer is 1.4741, which forms the 2% refractive index difference between the core and claddings. The height and width of single-mode waveguide is 4 µm × 4 µm under a 2% refractive index difference, which offers a relatively small waveguide and compact device size on the silica platform. The thicknesses of upper cladding, lower cladding, and core layer are 20 μm, 10 μm, and 4 μm, respectively. The effective refractive index as a function of dual-mode waveguide width at 1550 nm is shown in Figure 2. To support the E_11_ and E_21_ modes, the width of core waveguide *W*_0_ is set to be 9.8 μm, at which the effective refractive indices of E_11_ and E_21_ modes are 1.4654 and 1.4601, respectively. To facilitate the test, an asymmetric directional coupler (ADC)-based E_11_ and E_21_ mode multiplexer and demultiplexer was introduced at the input and output ports.

At the input end of the switch, a 1 × 2 MMI coupler is adopted to implement the dual-mode 3 dB power splitting for both E_11_ and E_21_ modes. As shown in Figure 1b, the width of tapered waveguide ranges from *W*_0_ = 9.8 μm to *W*_1_ = 12 μm, which is favorable to the gradual mode evolution. The conical waveguide holds a lower loss for the length *L*_0_ = 250 μm. The input waveguide center is spaced at *D*_MMI_ = 12 μm distance from the MMI center. According to the self-imaging principle [16,17], with a properly selected MMI coupler length, the 3 dB power splitting for two E_11_ and E_21_ modes can be achieved. The beat length *L*_π_ is the length that satisfies the 3 dB power splitting, which can be calculated by Equation (1) [18]:

(1) $$L_{\pi }=\frac{\pi }{\beta_{0}-\beta_{1}}=\frac{4n_{r}W_{MMI}^{2}}{3\lambda_{0}}$$

where *β*_0_ and *β*_1_ are the propagation constants of E_11_ and E_21_ modes, respectively; *n*_r_ is the corresponding effective refractive index; *W*_MMI_ is the multimode waveguide width; and *λ*_0_ is the operating wavelength. The detailed structure parameters of the dual-mode MMI coupler are optimized by BPM calculations. Therefore, the multimode waveguide length *L*_MMI_ and width *W*_MMI_ are set to be 2730 μm and 36 μm, respectively.

The conventional S-bend waveguide produces a large bending loss and mode mismatch in the case of small bending radius. By coupling the E_11_ and E_21_ modes into the trapezoidal waveguide on both sides through the directional coupler (DC) structure, the dual-mode S-bend waveguide proposed in this paper isolates two light beams while passing through the bending region. Since the optical fields of E_11_ and E_21_ modes are different in phase after coupling, these two modes can be coupled back to the original modes after the propagation through two parallel and equal-width bending waveguides. This structure is favorable to restrain the intermode crosstalk and bending loss, especially at a small bending radius.

As shown in Figure 1c,d, the width of the input tapered waveguide shrinks from *W*_DC0_ = 12 μm at the output position of MMI coupler to *W*_DC1_ = 1 μm at the top end. The length of L_DC0_ is 1227 μm. The distance *D*_0_ between the tapered waveguide and two narrow-to-wide tapered waveguides is 1 μm. And the width of narrow-to-wide tapered waveguide is widened from *W*_DC1_ = 1 μm to *W*_DC2_ = 4.9 μm, with a length of *L*_DC1_ = 870 μm. The radius of the bending waveguide is *R* = 4640 μm, while the bending angle is *θ* = 7.9°. The gap between two paralleled bending waveguides is *D*_1_ = 3 μm. At the output ending, the same structure and geometric parameters are adopted, except for the narrowing width. To reduce the optical loss, the width *W*_DC1_ and *W*_DC3_, the length *L*_DC2_ of central tapered waveguide is chosen to be 1 μm, 9.8 μm, and 982 μm, respectively.

According to the working principle of proposed thermo-optic switch, when no metal heater is working, the light will emerge from port Output 1. When the metal heater is working, the light will output from port Output 2. The refractive index change versus transmitted mode power can be studied by BPM calculations. By Equations (2) and (3), the temperature change and electrical driving power in MZI arm can be obtained [19,20]:

(2) $$\varepsilon =\frac{\Delta n}{\Delta T}$$

(3) $$P=L_{e}W_{e}K(1+0.88\frac{H}{W_{e}})\frac{\Delta T}{H}$$

where *ε* is the thermo-optic coefficient, Δ*n* is the refractive index change, Δ*T* is the temperature change, *L*_e_ is the electrode length, *W*_e_ is the electrode width, *K* is the thermal conductivity, *H* is the electrode-to-waveguide core layer height, and *P* is the driving power. Due to the very limited thermo-optic coefficient difference between the silica core and cladding, the thermo-optic coefficients are both chosen to be 1.19 × 10^−5^. The thermal conductivity *K* is 1.3 W∙m^−1^. The height *H* of electrode to waveguide core layer is 16 μm. According to the design rules of SiO_2_ platform, the electrode length *L*_e_ and width *W*_e_ are set to be 3000 μm and 21 μm, respectively.

In order to prevent thermal crosstalk, the spacing *D*_e_ between two arms is selected to be 200 μm. To further eliminate potential thermal crosstalk and improve the modulation efficiency, the air gap structure is adopted in fabrication. As shown in Figure 1c, the width *W*_Air_ and length *L*_Air_ of the air gap are 35 μm and 3000 μm, respectively. Another 5 μm thick silicon substrate is removed to better inhibit the thermal diffusion. Since the thermal conductivity of air is much smaller than that of silica and silicon, the thermal field can be better restrained within the arm to produce a more remarkable refractive index variation and resulting optical phase change. The extinction ratio and crosstalk of can be calculated by Equations (4) and (5):

(4) $$ER=\min\{10\log_{10}\frac{P_{\mathrm{target}}}{P_{\mathrm{other}-\mathrm{target}}}\}$$

(5) $$CT=\max\{10\log_{10}\frac{P_{\mathrm{other}}}{P_{\mathrm{target}}}\}$$

where *P*_target_ denotes the optical power output from the target port, *P*_other-target_ denotes the optical power remained in the target port, and *P*_other_ denotes the optical power output from the non-target port.

**Figure 3:**
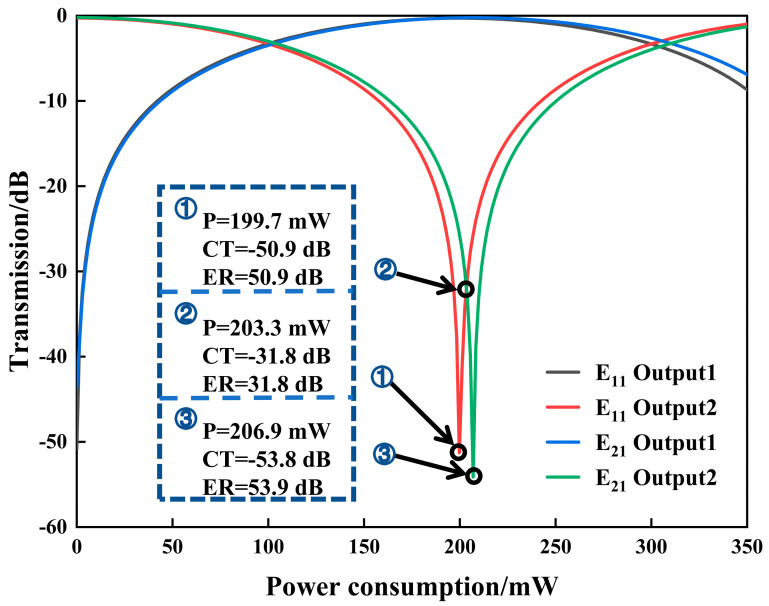
Transmittance of E_11_ mode and E_21_ mode versus electrical driving power at two output ports.

**Figure 4:**
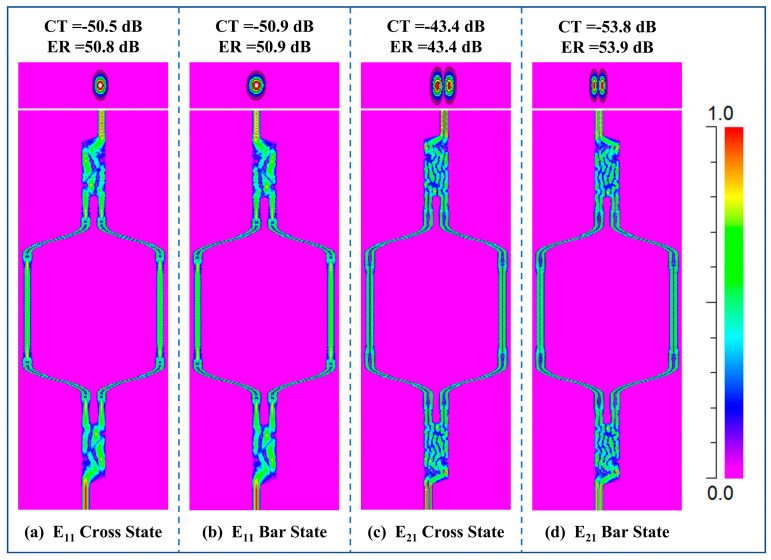
Mode field distribution of (**a**) E_11_ mode output from port Output2 (Cross state), (**b**) E_11_ mode output from port Output1 (Bar state), (**c**) E_21_ mode output from port Output2 (Cross state), (**d**) and E_21_ mode output from port Output1 (Bar state).

Based on the above device structure, the transmittance of the dual-mode thermo-optic switch as a function of the driving power is calculated, as shown in Figure 3. When E_11_ mode at *λ* = 1550 nm is coupled into the Input port and no driving power is applied, the transmittance at port Output2 is −0.3 dB. ER and CT are 30.1 dB and −50.5 dB, respectively. The corresponding mode field distribution is shown in Figure 4a. When a driving power of 199.7 mW is applied on the metal Electrode, the refractive index difference ∆*n* between two MZI arms is 0.000284. In this case, a π phase difference between two MZI arms is produced after the light passes through them. Under this condition, when the light continues propagating through the second MMI coupler, it outputs from port Output1. The transmittance at port Output1 is −0.2 dB. ER and CT are 50.98 dB and −50.9 dB, respectively. The mode field distribution in this case is shown in Figure 4b.

When the E_21_ mode at *λ* = 1550 nm is input and no metal heater is working, no refractive index difference between two arms exists. The transmittance at port Output2 is −0.2 dB. ER and CT are 33.5 dB and −43.4 dB, respectively. The mode field distribution is shown in Figure 4c. In case the Electrode is driven by a power of 206.9 mW, the transmittance at port Output1 is −0.2 dB. The corresponding ER and CT values are 53.88 dB and −53.91 dB, respectively. The mode field distribution is shown in Figure 4d.

Specifically, at the driving power of 203.3 mW, E_11_ mode and E_21_ mode exhibit the same transmittance, and almost the same ER and CT performance. The relatively high driving powers above are mainly due to the lower thermo-optic coefficient of silica.

**Figure 5:**
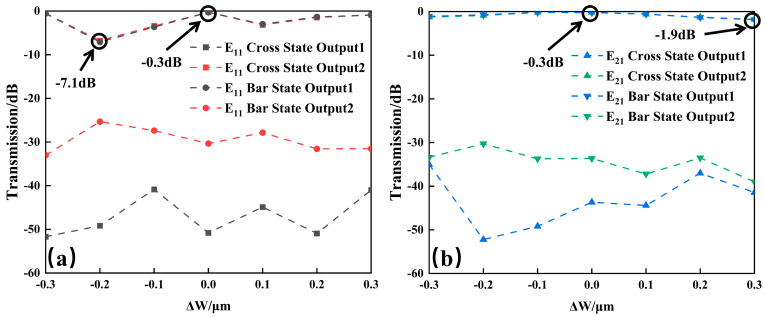
Transmission as a function of waveguide width change for (**a**) E_11_ mode and (**b**) E_21_ mode.

Fabrication tolerance is one of the critical issues that have an impact on performance. In this work, the main fabrication error comes from the photolithography and plasma etching-induced waveguide width deviation from the theoretical expectations. In practical fabrication, all waveguide widths are supposed to change at the same time. The effect of this width change on the optical transmission is theoretically investigated. Here, we set ΔW as the waveguide width change. When ΔW is positive, the waveguide width is larger than the theoretical value. When ΔW is negative, the waveguide width is smaller than the theoretical value. The optical transmission of this 1 × 2 switch as a function of ΔW is studied, as shown in Figure 5 below. The results show that the transmission loss increases by up to 6.8 dB for the E_11_ mode switching when ΔW is ±0.3 μm. The transmission loss change is less than 1.6 dB for the E_21_ mode switching when ΔW is ±0.3 μm. This is mainly due to the small coupling space in the DC coupling and S-bend region. The dimensional change will lead to over-coupling or under-coupling, which greatly affect the transmission of the E_11_ mode. Due to the relatively larger size of the E_21_ mode, its transmission is less sensitive to the waveguide width change.

## 3. Results and Discussion

The proposed dual-mode thermo-optic switch was fabricated on a silica platform (Shijia Ltd., Hebi, China). Firstly, a 10 μm thick low-refractive index silica lower-cladding was grown on the clean silicon substrate by thermal oxidation. Then, another 4 μm thick high refractive index germanium-doped silica was formed on the lower-cladding by plasma-enhanced chemical vapor deposition (PECVD) as the core layer. By ultraviolet (UV) photolithography and inductively coupled plasma etching (ICP), waveguide patterns were transferred to the core layer. Thereafter, another 20 μm thick low-refractive-index silica film was formed by PECVD on the core waveguide layer as the upper-cladding. After preparing the metal electrodes, another UV lithography and ICP etching were adopted to form the 35 μm deep air trenches on both sides of the metal electrodes.

**Figure 6:**
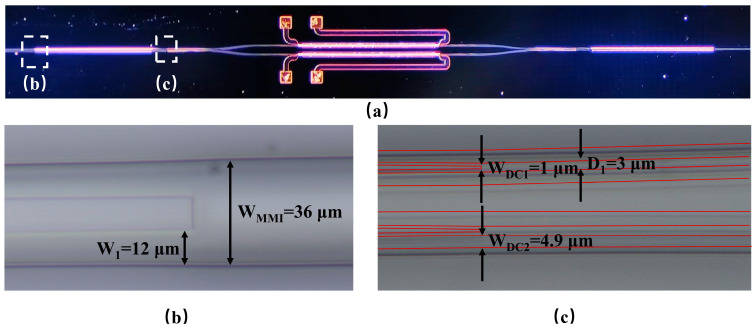
Optical microscope images of (**a**) prepared mode switch. Images (**b**,**c**) present the details of input waveguide of 1 × 2 MMI and the input waveguides of dual-mode S-bend, respectively.

**Figure 7:**
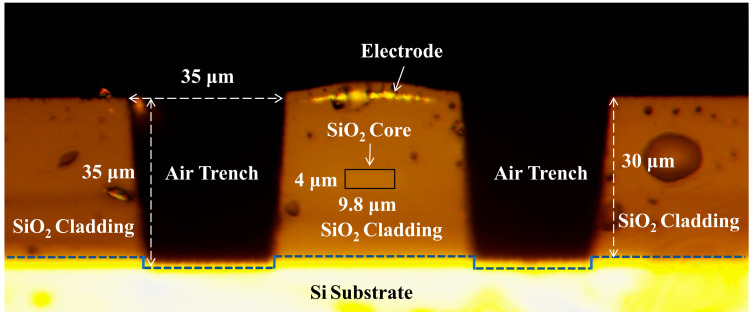
Cross-sectional view of air-trenched modulation arm.

Figure 6a shows the optical microscope images of prepared mode switch. Figure 6b,c present the details of input waveguide of 1 × 2 MMI and the input waveguides of dual-mode S-bend, respectively. The cross-sectional view of modulation arm that corresponds to Figure 1e is shown in Figure 7. As shown in the figure, the width and height of silica core waveguide are 9.8 μm and 4 μm, respectively. The 30 μm thick upper-cladding and lower-cladding besides the active arm was removed by ICP etching to form the air trench structure. The metal electrode is protected by an inert film. The implemented structural dimensions approximately coincide with designed values.

**Figure 8:**
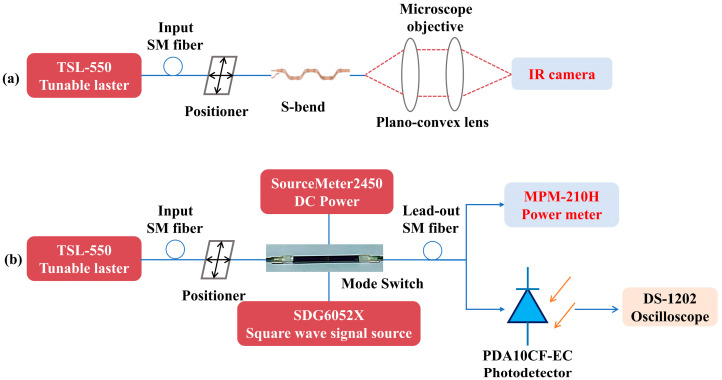
Block diagram of setup for the characterization of (**a**) S-bend, and (**b**) dynamic response of mode switch.

After dicing, the switch chip was firstly polished to smooth the facets. The prepared device was then characterized by the measurement setup shown in Figure 8. As mentioned above, an ADC-based E_11_–E_21_ mode (de)multiplexer has been introduced at the input and output ports. The light from a tunable laser (TSL-550, Santec Inc., Komaki, Japan) was selectively coupled into the mode multiplexer at the input end to excite the E_11_ or E_21_ mode. The output mode patterns were recorded by an infrared camera (7290A, MiconViewer Ltd., Fairfield, CA, USA), as shown Figure 8a. The rectangular electrical driving signal from functional signal generator (DG4000, Rigol Ltd., Suzhou, China) was applied on the metal phase shifter through the contract with pads. DC power supply (SourceMeter2450, Keithley, Solon, OH, USA) was used to adjust the initial phase difference between two arms. The optical power was recorded by the optical power meter (MPM-210, Santec Inc., Komaki, Japan). The dynamic response was characterized by an oscillator (DS1202, Rigol Ltd., Suzhou, China). Before feeding into the oscilloscope, the optical output signal was firstly collected by an InGaAs photodetector (PDA10CF-EC, Thorlabs Inc., Newton, NJ, USA), as shown in Figure 8b.

**Figure 9:**
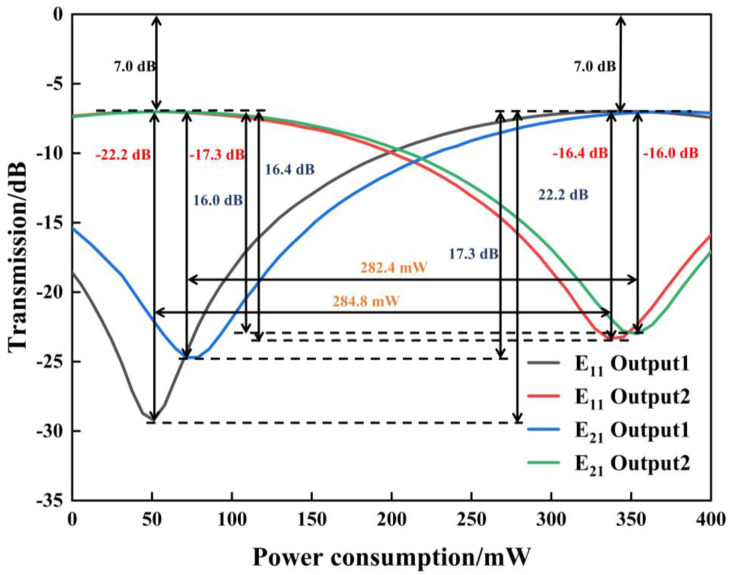
Dual-mode thermo-optic switch electrode drive power plotted as a function of transmittance, including inputs E_11_ mode and E_21_ mode.

With the measurement setup in Figure 8, the switching performance of the dual-mode switch is characterized at *λ* = 1550 nm and shown in Figure 9. Firstly, the E_11_ mode is input into the 1 × 2 MMI coupler. When an electrical driving power of 284.8 mW is applied on the phase shifter, the output E_11_ mode switches from Output2 to Output1 (Cross state to Bar state). ERs at Output1 and Output2 are 22.2 dB and 16.4 dB, respectively. The mode CT between Output1 and Output2 is −16.4 dB. Then, the E_21_ mode is input into the 1 × 2 MMI coupler. When an electrical driving power of 282.4 mW is applied on the phase shifter, the output E_21_ mode switches from Output2 to Output1 (Cross state to Bar state). The ERs at Output1 and Output2 are 17.3 dB and 16.0 dB, respectively. The mode CT between Output1 and Output2 is −16.0 dB.

The insertion loss (IL) around 7 dB at Bar state and Cross state mainly includes the loss caused by the fiber–waveguide coupling, mode conversion in the (de)multiplexer, S-bends, and MMI couplers. On the 2% refractive index difference planar lightwave circuit (PLC) platform, the propagation loss of the single-mode silica waveguide is 0.12 dB/cm. The optical loss induced by the coupling of a single-mode fiber (SMF) with a silica waveguide edge coupler is about 0.4 dB/facet [21]. Since the E_11_–E_21_ mode (de)multiplexer is the same as that we have demonstrated in Ref. [22], the loss induced by the mode multiplexer is shown to be 1.79 dB and 1.91 dB for the E_11_ mode and E_21_ mode, respectively.

**Figure 10:**
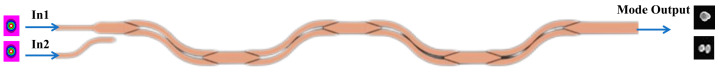
Schematic diagram of four cascaded S-bends with ADC mode multiplexer for mode pattern characterization.

The investigation of the S-bend mainly focuses on the optical loss of the E11 mode and E21 mode when they pass through the S-bend. However, in the complete device structure, the optical loss of one single S-bend is hard to be separated from the total loss. In addition, the losses induced by two MMI couplers play a key role during the loss characterization. To well investigate the E_1l_ and E_2l_ mode propagation along the proposed dual-mode S-bend, four S-bends with the same size to that adopted in the mode switch were cascaded to evaluate the optical performance. As shown in Figure 10, an ADC-type E_1l_ and E_2l_ mode multiplexer is placed at the input end. A 9.8 um-wide multimode waveguide that supports the E_2l_ mode is used at the output end to observe the mode pattern. When the E_1l_ mode is coupled into the port In1, it will propagate along the cascaded S-bends without mode evolution and output from the multimode waveguide. The captured fundamental mode pattern is shown in Figure 10. When the E_1l_ mode is coupled into the In2 port, the E_2l_ mode will be excited by the mode multiplexer. After passing through the cascaded S-bends, the E_2l_ mode is supposed to be observed at the output end. The captured E_2l_ mode pattern is clearly presented in Figure 10. Based on the sectional loss mentioned above, the structural loss of the single S-bend can be deduced by Equation (6):

(6) $$IL_{S-Bend}=\frac{IL_{Test}-PL-CL-IL_{(de)multiplexer}}{4}$$

Thereby, the loss values of the S-bend are 0.59 dB and 0.23 dB for the E_1l_ mode and E_2l_ mode, respectively.

**Figure 11:**
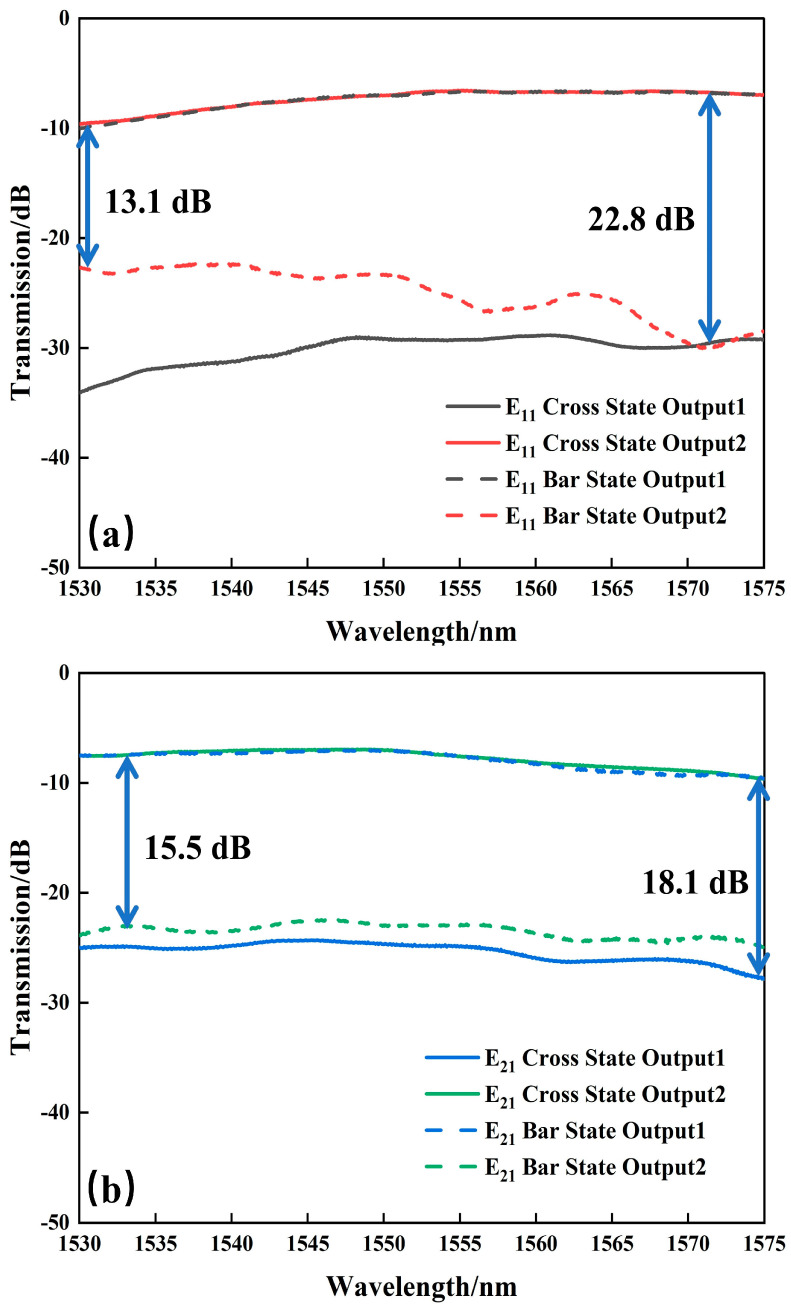
Transmittance versus (**a**) E_11_ mode and (**b**) E_21_ mode.

The bandwidth characteristics of the fabricated switch are characterized, as shown in Figure 11. The measurement results show that within the wavelength range from 1530 to 1575 nm, for the E_11_ mode input, the switch exhibits the ER of ≥13.1 dB and CT ≤ −22.8 dB at an electrical driving power of 284.8 mW, as shown in Figure 11a. Meanwhile, for the E_21_ mode input, the ER is ≥15.5 dB and CT is ≤−18.1 dB at an electrical driving power of 282.4 mW, as shown in Figure 11b.

**Figure 12:**
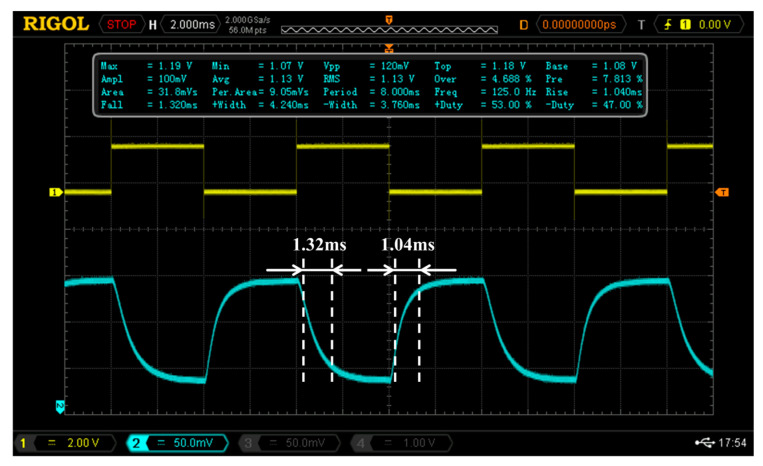
Dual-mode thermo-optic switch response time graph.

The time response of the fabricated mode switch is shown in Figure 12 below. When the 125 Hz rectangular driving signal is applied on the electrode, the measured rise time and fall time are 1.04 ms and 1.32 ms, respectively. This relatively large response time is mainly limited by the physical characteristic of thermo-optic modulation that relies on the dielectric relaxation and resulting refractive index variation. Reducing the path length for thermal diffusion will be favorable to decrease the response time.

The performance comparison between the reported works and the presented dual mode switch is summarized in Table 1. Most mode switches are based on the polymer waveguide that hold similar refractive index difference to that of the silica waveguide. Since the thermo-optic coefficient of polymer commonly is one order higher than that of silica, the driving power of silica waveguide switch is much higher. The relative longer response time also could be attributed to the 20 μm thick upper cladding, which apparently implies a large thermal diffusion distance. Except for that, close ER and CT values can be observed. The lower IL obtained in this work is mainly due to the optimized design and precise fabrication, which offers good dimension matching between the designed waveguide and the prepared device. In fact, the most favorable aspect of this silica switch is the relatively wide bandwidth, which is largely due to the adoption of MMI couplers.

## 4. Conclusions

A silica waveguide dual-mode 1 × 2 thermo-optic switch is theoretically designed and experimentally demonstrated. An MMI coupler combined with a Mach–Zehnder interferometer is adopted to implement the E_11_ and E_21_ mode selective output. Small-radius bimodal S-bends allow the propagation of dual modes. After BPM design optimization and CMOS compatible fabrication, the switching performance levels are characterized in detail. Within the wavelength range from 1530 nm to 1575 nm, for the E_11_ mode input, the measured extinction ratio (ER) is ≥13.1 dB and the crosstalk (CT) is ≤−13.1 dB at the electrical driving power of 284.8 mW. Meanwhile, for the E_21_ mode input, the measured ER is ≥15.5 dB and CT is ≤−15.5 dB at the electrical driving power of 282.4 mW. Compared with reported works with limited refractive index differences, the proposed switch presents a relatively wide bandwidth, smaller optical loss, and compact footprint by the introduction of bimodal S-bends and MMI couplers. Though the power consumption and response time are limited by the intrinsic low thermo-optic coefficient of silica, by the design optimization of cladding layers, such as the introduction of an air gap and cladding thickness reduction, better power performance can be expected. The demonstrated switch has good potential in on-chip MDM applications.

## Figures and Tables

**Table 1:** Switching performance comparison with reported works.

Ref	Material	Size /μm	Power /mW	IL /dB	ER /dB	CT /dB	Speed /ms	BW /nm
[23]	Polymer	30,000	9.0	−11.3	16.4	−16.4	1.34/1.32	-
[24]	Polymer	16,500	52/129	−10/−11	10	-	0.3/0.4	35
[25]	Polymer	27,000	20.6/22.6	−9.2	15.6/14.1	−15.6	4.4/2.9	35
[26]	Polymer	25,000	10	−8.9	14	-	3.7	35
[27]	Polymer	22,500	128	-	13	-	0.824/0.944	35
This	Silica	16,000	284.8/282.4	−7.0	16.0	−15.9	1.04/1.32	45

## Data Availability

Data is contained within the article.
